# Successful management of severe pulmonary form of leptospirosis with VV-ECMO, prone ventilation, and bronchoalveolar lavage: two case reports

**DOI:** 10.3389/fmed.2025.1598589

**Published:** 2025-05-30

**Authors:** Meisong Chen, Haohao Wu, Lutao Xie, Min Wu, Pin Lan

**Affiliations:** Department of Emergency Medicine, Lishui Central Hospital, The Fifth Affiliated Hospital of Wenzhou Medical University, Lishui, China

**Keywords:** leptospirosis, severe pulmonary form of leptospirosis (SPFL), diffuse alveolar hemorrhage (DAH), VV-ECMO, ARDS, mNGS (metagenomic next-generation sequencing), case report

## Abstract

**Background:**

Leptospirosis is a globally prevalent zoonotic acute infectious disease that can rapidly progress to severe pulmonary form of leptospirosis (SPFL), leading to multiple organ failure with a high mortality rate. It is estimated that approximately 58,900 deaths occur annually due to leptospirosis, with critically ill patients admitted to intensive care units facing extremely high fatality rates. Therefore, timely and effective treatment strategies are crucial.

**Case presentation:**

Two patients developed fever after farm work exposure, followed by progressive dyspnea and hemoptysis, leading to hospitalization. They rapidly developed acute respiratory distress syndrome (ARDS) and diffuse alveolar hemorrhage (DAH) with severe thrombocytopenia, accompanied by a continuous decline in the ratio of the partial pressure of arterial oxygen to the fraction of inspired oxygen (PaO₂/FiO₂ [P/F]). Despite endotracheal intubation and mechanical ventilation, hypoxemia persisted. Venovenous extracorporeal membrane oxygenation (VV-ECMO) was initiated to provide oxygenation support, heparin anticoagulation was not used in the early stage. Meanwhile, prone ventilation and bronchoscopy alveolar lavage were performed to promote the clearance of pulmonary hemorrhage, along with anti-infection treatment. The diagnosis of leptospirosis was confirmed through Metagenomic Next-Generation Sequencing (mNGS). Both patients ultimately recovered, were successfully weaned from life support, discharged in stable condition, and returned to normal life.

**Conclusion:**

Early VV-ECMO support, combined with prone ventilation and bronchoalveolar lavage, can improve the prognosis of patients with SPFL. mNGS testing aids in the definitive diagnosis of leptospirosis and provides a reliable basis for antibiotic selection.

## Background

Leptospirosis is a globally prevalent zoonotic acute infectious disease caused by bacteria of the genus *Leptospira*, with an estimated 1.03 million new cases and 58,900 deaths annually worldwide. The majority of cases and fatalities occur in adult males aged 20–49 years. Clinical manifestations vary widely, ranging from mild flu-like symptoms to severe multiple organ dysfunction syndrome, including pulmonary hemorrhage syndrome and acute hepatorenal injury (1). Between 20 and 70% of patients with leptospirosis progress to SPFL, which is characterized by rapid deterioration, uncontrolled pulmonary hemorrhage, and refractory hypoxemia, ultimately leading to DAH and severe ARDS—the primary causes of mortality in leptospirosis (2, 3).

VV-ECMO, a life-support technology that serves as a temporary substitute for pulmonary function, has shown potential efficacy in treating DAH. A systematic review indicated that ECMO-supported DAH patients might achieve favorable short-term survival rates (4). Advancements in modern ECMO technology have enabled lower anticoagulation strategies to maintain circuit patency while minimizing bleeding risks. Concurrently, the emergence of molecular diagnostic techniques has highlighted the role of mNGS in rapid and precise pathogen identification, particularly in cases of unexplained or complex infections. This technology provides critical guidance for early antibiotic selection and therapy (5).

This report aims to explore the role of early VV-ECMO support combined with prone ventilation and bronchoscopy-guided alveolar lavage in the management of SPFL. Additionally, it discusses the diagnostic value of mNGS in detecting leptospiral and other rare pathogen infections, sharing our clinical treatment experience.

## Case presentation

### Case 1

**Figure 1:**
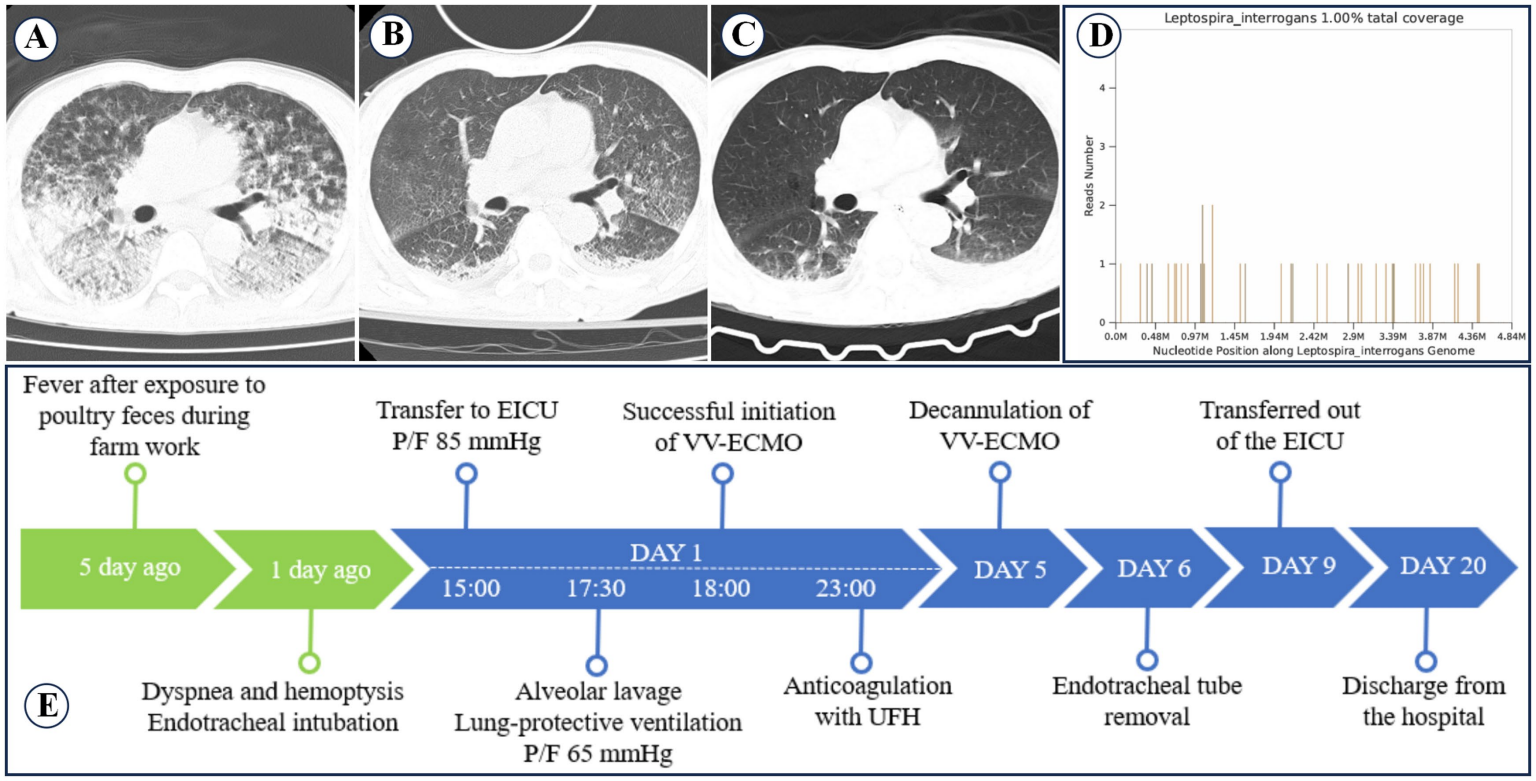
Changes in pulmonary imaging and treatment process of case 1. **(A)** Chest CT scan at the local hospital shows diffuse infiltrative lesions in both lungs. **(B)** Chest CT scan after VV-ECMO removal shows mild interstitial pneumonia in both lungs. **(C)** Chest CT before discharge shows significant improvement in both lungs. **(D)** mNGS detected *Leptospira interrogans* with a coverage rate of 1.00%. **(E)** Treatment process of Case 1.

A 69-year-old male farmer with no prior medical history was admitted to a local hospital after experiencing fever for 5 days and dyspnea with hemoptysis for 1 day. He had a history of exposure to poultry manure while working on his farm 5 days prior. Chest computed tomography (CT) revealed pulmonary infection and interstitial changes (Figure 1A). The primary diagnosis was severe pneumonia and acute respiratory failure, and the patient was placed on mechanical ventilation via endotracheal intubation. However, his oxygen saturation continued to deteriorate, and bloody sputum was aspirated from his airway. Twenty hours after intubation, he was transferred to our emergency intensive care unit (EICU).

Upon arrival at the EICU, the patient was in a sedated and analgesic state, with a body temperature of 36.8°C, respiratory rate of 17 breaths/min, pulse rate of 138 beats/min, and blood pressure of 137/90 mmHg (with norepinephrine infusion at 0.5 μg/kg/min). Oxygen saturation was 88%. He remained on mechanical ventilation in volume-controlled mode with a tidal volume of 350 mL, an FiO₂ of 100%, and a positive end-expiratory pressure (PEEP) of 15 cmH₂O (1 mmH₂O = 0.098 kPa). A large amount of fresh red bloody sputum was aspirated from the airway, and extensive moist rales were heard in both lungs. Laboratory results showed: P/F, 85 mmHg; Platelet count (PLT), 43 × 10^9^/L; C-reactive protein (CRP), 213.74 mg/L; Procalcitonin (PCT), >100 ng/mL; Activated partial thromboplastin time (APTT), 36.8 s; Myoglobin (MYO), >2000 ng/mL; Creatinine (CR), 240 mg/dL; Alanine aminotransferase (ALT), 111 U/L; Total bilirubin (TBIL), 70 μmol/L. The primary diagnosis included severe pneumonia, DAH, severe ARDS, septic shock, and hepatic and renal dysfunction.

Despite optimized mechanical ventilation, his P/F remained below 80 mmHg for over 3 h. VV-ECMO and CRRT support were initiated, along with intermittent prone ventilation. Mechanical ventilation was switched to pressure control mode (inspiratory pressure 12 cmH₂O, tidal volume 150–350 mL, PEEP 15 cmH₂O, plateau pressure ≤30 mmHg). Empirical antibiotic therapy included piperacillin-tazobactam (4.5 g every 8 h) combined with omadacycline (100 mg daily, initial dose 200 mg IV). The patient also received methylprednisolone (200 mg IV) for anti-inflammatory treatment, recombinant human thrombopoietin (15,000 U SC daily) to increase platelet count, platelet transfusion (10 U), fresh plasma, packed red blood cells, and norepinephrine (0.1–0.8 μg/kg/min) for blood pressure support. Bronchoscopic alveolar lavage was performed, and both blood and lavage fluid were sent for mNGS testing. Unfractionated heparin (UFH) anticoagulation was initiated at the 5th hour of VV-ECMO operation. On day 3, mNGS confirmed *Leptospira interrogans* infection, with a coverage rate of 1% and a relative abundance of 16.45% (Figure 1D). On day 5, VV-ECMO was discontinued, and omadacycline was stopped. Follow-up chest CT showed improvement in pulmonary hemorrhage compared to before (Figure 1B). The endotracheal tube was removed on day 6, the patient was transferred out of the EICU on day 9, and was discharged after recovery on day 20. A follow-up chest CT before discharge showed that pulmonary hemorrhage had significantly improved (Figure 1C). The treatment process is summarized in Figure 1E, and the trends in blood test results are detailed in Table 1. Changes in ventilator parameters, ECMO parameters, and medication dosages during ECMO are detailed in Table 2.

**Table 1:** Changes in laboratory test results for case 1.

TIME	PLT (×10^9^/L)	CRP (mg/L)	PCT (ng/mL)	APTT (s)	MYO (ng/mL)	CR (mg/dL)	ALT (U/L)	TBIL (μmol/L)
DAY 1	43	213.74	>100	36.8	>2000	240	111	70.2
DAY 4	68	50.16	43.07	41.5	293.8	131	58	60.7
DAY 5	77	28.91	11.71	49.2	117.6	115	72	70.4
DAY 6	50	51.82	4.79	51.1	193.6	104	104	67.1
DAY 8	177	38.63	2	28.3	381.6	129	91	39.5
DAY 10	468	34.04	0.52	29.6	151.1	116	81	42.7
DAY 20	443	13.36	0.07	29.9	–	105	35	15.7

**Table 2:** Changes in ventilator parameters, ECMO parameters, and medication dosages during ECMO.

	Case 1						Case 2					
	Day1	Day2	Day3	Day4	Day5	Day6	Day1	Day4	Day6	Day7	Day9	Day11
Ventilatory parameters												
Respiratory rate	12	12	12	12	14	17	12	12	12	14	16	18
Ventilation mode	PC	PC	PC	PC	PC	PC	VC	VC	VC	PC	PC	PC
Pi (cmH2O)	12	10	10	12	12	12	-	-	-	14	12	12
FiO_2_ (%)	40	30	35	35	40	35	35	30	30	40	45	45
PEEP (cmH_2_O)	15	15	12	12	12	8	20	18	15	12	10	8
Tidal volume (mL)	320	340	350	330	550	650	240	300	300	450	600	600
Oxygen saturation (%)	93	92	95	96	96	93	96	93	92	95	97	92
ECMO record												
Sweep gas flow (L/min)	3.5	3.5	3.5	3.5	3.5	0	3.5	3.5	3.5	3.5	3	0
ECMO flow (L/min)	3.38	3.3	3.37	3.35	3.4	2	3.35	3.48	3.38	3.35	3.15	2
UFH (U/h)	0–125	175	500	750	500	500	0	0–150	200	250	500	600
Methylprednisolone (mg/d)	200	200	200	80	80	40	500	200	80	40	40	40

### Case 2

**Figure 2:**
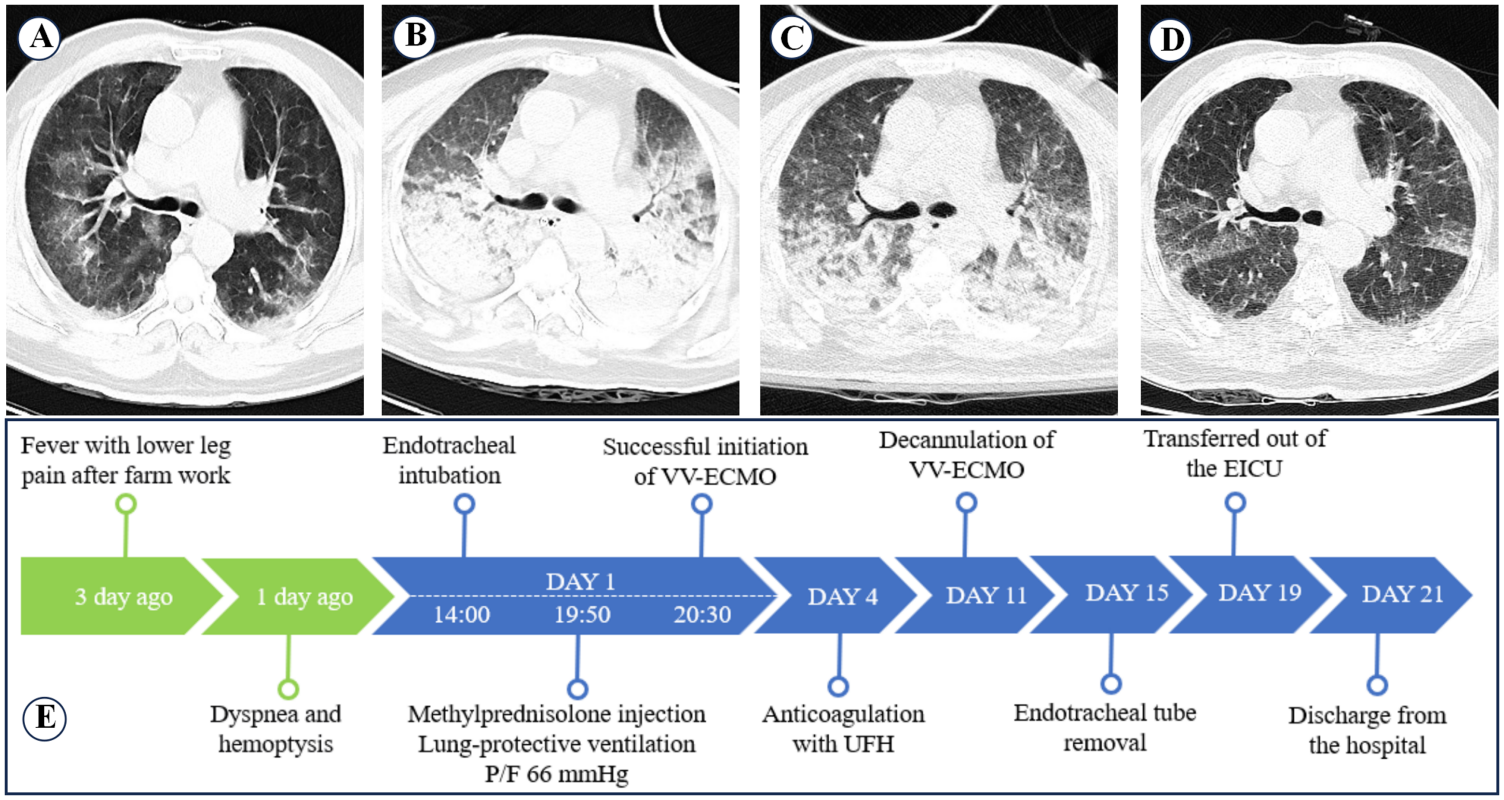
Changes in pulmonary imaging and treatment process of case 2. **(A)** Chest CT scan at admission shows scattered diffuse infiltrative lesions in both lungs. **(B)** Chest CT scan on the 4th day of hospitalization shows diffuse infiltrative lesions and atelectasis in both lungs. **(C)** Chest CT scan after VV-ECMO removal shows improvement in diffuse infiltrative lesions and atelectasis in both lungs. **(D)** Chest CT before discharge shows significant improvement in both lungs. **(E)** Treatment process of Case 2.

A 72-year-old male farmer with no prior medical history presented to our emergency room with fever and bilateral calf pain for 3 days, followed by hemoptysis for 1 day. Upon arrival at the emergency room, the patient experienced respiratory distress and orthopnea, with a body temperature of 38.6°C, respiratory rate of 30 breaths/min, pulse rate of 129 beats/min, and blood pressure of 129/70 mmHg. With an oxygen mask at 8 L/min, the oxygen saturation was 89%. Chest CT revealed bilateral pulmonary exudative changes (Figure 2A). His initial lab results were: P/F, 124 mmHg; PLT, 12 × 10^9^/L; CRP, 219.72 mg/L; PCT, 74.68 ng/mL; APTT, 30 s; MYO, >2000 ng/mL; CR, 128 mg/dL; ALT, 98 U/L; TBIL, 64.1 μmol/L. The primary diagnosis included severe pneumonia, DAH, severe ARDS, and hepatic and renal dysfunction.

The patient was intubated and mechanically ventilated with 100% FiO₂ support, but his P/F declined to 66 mmHg. Emergency VV-ECMO and CRRT support were initiated, along with intermittent prone ventilation. He remained on volume-controlled ventilation (tidal volume 200–300 mL, PEEP 20 cmH₂O, plateau pressure ≤35 mmHg). Empirical antibiotic therapy included moxifloxacin (0.4 g IV daily) combined with imipenem/cilastatin (1 g IV every 8 h). The patient also received methylprednisolone (500 mg IV for 3 days), recombinant human thrombopoietin (15,000 U SC daily), fresh plasma, packed red blood cells, norepinephrine (0.1–1.2 μg/kg/min), and bronchoscopic alveolar lavage. Both blood and lavage fluid were sent for mNGS testing. On day 3, mNGS confirmed leptospirosis, and platelet transfusion (10 U) was administered on day 4, but a follow-up chest CT showed worsening pulmonary hemorrhage (Figure 2B). On day 11, VV-ECMO was discontinued, and a follow-up chest CT showed significant improvement in pulmonary hemorrhage (Figure 2C). The total duration of VV-ECMO support was 255 h, with the first 60 h without UFH anticoagulation. On day 15, the endotracheal tube was removed. On day 17, the patient was transferred out of the EICU. On day 21, the patient was discharged after recovery. A follow-up chest CT before discharge showed that pulmonary hemorrhage had significantly improved (Figure 2D). The treatment process is summarized in Figure 2E, and the trends in blood test results are detailed in Table 3. Changes in ventilator parameters, ECMO parameters, and medication dosages during ECMO are detailed in Table 2.

**Table 3:** Changes in laboratory test results for case 2.

TIME	PLT (×10^9^/L)	CRP (mg/L)	PCT (ng/mL)	APTT (s)	MYO (ng/mL)	CR (mg/dL)	ALT (U/L)	TBIL (μmol/L)
DAY 1	12	219.72	1.62	30	>2000	128	98	64.1
DAY 3	26	200.77	61.45	31.1	–	269	46	63.9
DAY 4	22	122.91	22.17	29.9	174.5	239	43	82.2
DAY 7	83	27.59	0.59	31.3	372	140	39	66.5
DAY 11	93	33.09	0.18	30.5	–	78	32	–
DAY 12	121	83.01	0.29	26.5	–	136	33	65.7
DAY 21	173	1.82	0.04	25.8	–	57	48	23.5

## Discussion

Leptospirosis is a globally prevalent zoonotic acute infectious disease caused by infection with Gram-negative aerobic spirochetes of the genus *Leptospira*. It progresses rapidly and has a high mortality rate. The disease is predominantly found in tropical and subtropical regions with humid and rainy climates. Transmission occurs through direct contact with the urine or reproductive secretions of infected animals or indirect contact with contaminated water or soil. It is particularly common among agricultural workers and outdoor enthusiasts, while urban cases requiring hospitalization are relatively rare (1, 6). Leptospirosis follows a biphasic clinical course. The bacteremic phase occurs within 5–7 days post-infection, during which bacteria spread through the bloodstream. Symptoms such as sudden fever, chills, myalgia, and headache appear in 75–100% of patients, with most cases being self-limiting and deaths being rare in this stage. The subsequent immune phase, occurring 4 to 30 days after symptom onset, is known as Weil’s disease, characterized by ARDS with DAH, worsening jaundice, acute kidney injury, and multi-organ dysfunction, leading to high mortality rates (1, 7). An estimated 58,900 deaths worldwide are attributed to leptospirosis, though the actual number may be higher due to underreporting in impoverished and non-surveillance regions (8). Studies have identified dyspnea, hypotension, and acute kidney injury as independent risk factors for ICU admission in leptospirosis patients, with ICU mortality exceeding 52% (1, 9).

Leptospirosis can be classified based on organ involvement into pulmonary hemorrhagic, jaundiced hemorrhagic, and renal forms. The pulmonary hemorrhagic form is further divided into mild and diffuse hemorrhagic types, with the latter being known as SPFL. SPFL is a severe pulmonary complication with high fatality rates, primarily caused by hypersensitivity reactions to *Leptospira* and its toxins, leading to capillary damage, extreme congestion, hemorrhage, and erythrocyte accumulation in the alveoli (10, 11). Leptospirosis is often underdiagnosed due to its non-specific clinical presentation, and definitive diagnosis is challenging. However, metagenomic next-generation sequencing (mNGS) has demonstrated significant advantages in pathogen detection, particularly in complex and unidentified infections. Studies have shown that mNGS has higher sensitivity (90%) and specificity (86%) in detecting *Leptospira* in blood samples compared to conventional microbiological tests. This enables timely antibiotic adjustments and improved patient outcomes (5, 12). Antibiotic therapy is the cornerstone of leptospirosis treatment. Empirical antibiotics should be initiated promptly when infection is suspected, with options including penicillins, macrolides, quinolones, or third-generation cephalosporins. However, clinicians should be vigilant about Jarisch-Herxheimer reactions, which occur in approximately 21% of patients after antibiotic administration and can exacerbate the disease course (13).

VV-ECMO is an extracorporeal life support technique that provides oxygenation support for severe respiratory failure, improving oxygenation in critical ARDS patients and reducing the 90-day mortality rate (14). Since SPFL rapidly progresses to severe ARDS, mechanical ventilation alone may be insufficient to maintain adequate oxygenation. VV-ECMO has been shown to be effective in improving oxygenation in cases where invasive mechanical ventilation fails. A systematic review found that ECMO-supported DAH cases have promising short-term survival rates (4). However, SPFL patients often present with severe thrombocytopenia, coagulopathy, and extensive pulmonary hemorrhage, increasing the risk of clot formation obstructing the airway. Therefore, ECMO anticoagulation management and airway blood drainage strategies are critical. ECMO has been successfully operated without systemic anticoagulation, with a median run time of 4.75 days (15). However, oxygenator thrombosis can compromise ECMO efficiency and worsen patient outcomes (16). Advances in ECMO membrane lung and circuit technology now allow for low-level anticoagulation strategies that maintain circuit patency while minimizing bleeding risks. For patients with active bleeding or high hemorrhage risk, anticoagulation strategies require careful balance. A meta-analysis by Lv et al. compared conservative anticoagulation strategies (APTT < 45 s or ACT 140–160 s) versus standard anticoagulation (APTT 50–70s or ACT 180–220 s), finding that gastrointestinal and surgical site bleeding rates were significantly lower in the conservative group, with no significant difference in thrombosis incidence (17). Some studies recommend maintaining APTT between 40 and 60 s for DAH cases requiring heparin anticoagulation (18). Both patients presented with massive hemoptysis and refractory hypoxemia, for which VV-ECMO support was initiated. To minimize the risk of oxygenator thrombosis, intravenous hemostatic agents were not administered. Although intratracheal administration of recombinant activated factor VII has been reported to be effective in managing pulmonary hemorrhage (19), this technique was not feasible in our center due to technical limitations. As a result, our management strategy involved withholding UFH during the early phase of ECMO support. In one case, the anticoagulation-free period lasted up to 160 h, with APTT maintained between approximately 30 and 50 s during ECMO operation.

In SPFL patients, DAH leads to increased airway pressure, necessitating careful control of ventilator driving pressure. Among ARDS patients with refractory hypoxemia receiving ECMO, driving pressure is the only ventilator setting independently associated with in-hospital mortality (20). Protective ventilation strategies on VV-ECMO recommend using the lowest possible tidal volume (4 mL/kg) and maintaining a plateau pressure ≤25 cmH₂O (21). Compared to low and moderate tidal volume mechanical ventilation, VV-ECMO combined with prone positioning improves survival rates (22). In both reported cases, the presence of severe hypoxemia, massive hemoptysis, and shock rendered prone positioning prior to ECMO initiation high-risk and potentially detrimental to timely resuscitation. VV-ECMO provides sufficient blood oxygenation, allowing for low driving pressure, low tidal volume lung-protective ventilation strategies combined with prone positioning and bronchoscopic alveolar lavage. This facilitates airway blood clearance, reduces bronchial clot formation, and minimizes the lung injury caused by mechanical ventilation.

## Conclusion

These two cases demonstrate the successful application of VV-ECMO combined with prone ventilation and bronchoalveolar lavage in the treatment of patients with SPFL. They also validate the safety and feasibility of an early heparin-free and goal-directed heparin reduction strategy. Additionally, they highlight the need for clinicians to be vigilant for leptospiral infection in patients who present with hemoptysis, severe ARDS, and a rapid decline in platelet count within a short period—especially those from endemic areas, rural settings, or with a history of exposure to animal excreta. Early mNGS testing is recommended for definitive diagnosis.

## Data Availability

The original contributions presented in the study are included in the article/supplementary material, further inquiries can be directed to the corresponding author.
